# A live-cell ergosterol reporter for visualization of the effects of fluconazole on the human fungal pathogen *Candida albicans*

**DOI:** 10.1128/mbio.02493-23

**Published:** 2023-11-30

**Authors:** Antonio Serrano, Miguel A. Basante-Bedoya, Martine Bassilana, Robert A. Arkowitz

**Affiliations:** 1 Université Côte d’Azur, CNRS, INSERM, Institute of Biology Valrose (iBV), Nice, France; The University of Texas Health Science Center at Houston, Houston, Texas, USA

**Keywords:** ergosterol, antifungal agents, *Candida albicans*, fungal growth, lipid signaling

## Abstract

Ergosterol, an essential plasma membrane amphipathic lipid, is a major component of the fungal plasma membrane. Most fungal pathogens are susceptible to azole drugs that target ergosterol biosynthesis, and resistance/tolerance to azoles is increasingly problematic. *Candida albicans* is the most prevalent etiology of candidiasis, and, in this fungal pathogen, ergosterol-rich sub-domains are likely to include sphingolipids, as well as specific membrane proteins, such as multidrug transporters. To investigate the dynamics of ergosterol during the cell cycle and whether drug treatment affects these dynamics in this opportunistic pathogen, we adapted the D4H (domain 4 of the perfringolysin O bacterial toxin) reporter for studying sterol-rich membrane domains. We show that D4H provides a direct readout for the cellular effects of fluconazole and that highly polarized ergosterol is not critical for budding or filamentous growth.

**IMPORTANCE:**

Ergosterol is a critical membrane lipid in fungi. In *Candida albicans*, this essential plasma membrane amphipathic lipid is important for interactions with host cells, in particular, host immune responses. Here, we use a live-cell reporter for specifically visualizing ergosterol and show that apical enrichment of this sterol is not critical for budding and filamentous growth in this human fungal pathogen. Our results highlight that this live-cell reporter is likely to be a useful tool in the analyses of azole resistance and tolerance mechanisms, including alterations in drug targets and upregulation of efflux activities.

## OBSERVATION

Most fungal pathogens are susceptible to azole drugs that target ergosterol biosynthesis. Resistance and tolerance to azoles are particularly problematic because these drugs are used extensively in medical, as well as agricultural, applications. The emergence of new drug-resistant fungal variants and species, including *Candida auris*, is a global concern recently recognized by the World Health Organization and the U.S. Centers for Disease Control (1, 2). The majority of sterols are found in the plasma membrane, with ergosterol making up >10 mol% of the *Saccharomyces cerevisiae* lipidome (3), and it is estimated to be approximately 40 mol% of the plasma membrane lipids (4). In *S. cerevisiae*, ergosterol is found predominantly in the inner leaflet of the plasma membrane, and roughly half of all lipids in this leaflet are sterols, approaching their solubility limit (5).

In fungi, ergosterol, an essential plasma membrane amphipathic lipid, is critical for membrane fluidity. *Candida albicans* is the most prevalent etiology of candidiasis, and, in this fungal pathogen, ergosterol-rich sub-domains are likely to include sphingolipids, as well as specific membrane proteins such as those involved in lipid metabolism and multidrug transporters (6). Interestingly, *C. albicans* mutants with reduced ergosterol levels trigger weaker host immune responses (7) and are defective in the lysis of host macrophages (8, 9). This suggests that sterols are important for interactions with host cells.

An important question is, what are the dynamics of ergosterol during the cell cycle and whether drug treatment affects these dynamics? To address this question, one must be able to visualize the distribution of ergosterol within the plasma membrane, which is challenging. Filipin, a fluorescent polyene macrolide antibiotic, has potent antifungal activity and induces membrane deformations and lesions (10, 11), limiting its use for live-cell imaging (12, 13). Recently, an improved version of the domain 4 of the perfringolysin O bacterial toxin (14) that binds free membrane sterols (but not sterols complexed with sphingolipids) down to a threshold of 20 mol %, D4H, was used to visualize ergosterol dynamics in the live cells of model yeasts *Schizosaccharomyces pombe* and *S. cerevisiae* (8, 15, 16). In this study, we adapted this D4H reporter assay for studying sterol-rich membrane dynamics in *C. albicans*, a human fungal commensal and opportunistic pathogen. We show that D4H provides a direct readout for the cellular effects of fluconazole (FCZ) in *C. albicans* and that highly polarized ergosterol is not critical for budding or filamentous growth in this organism.

### Ergosterol dynamics during *C. albicans* growth

**Fig 1:**
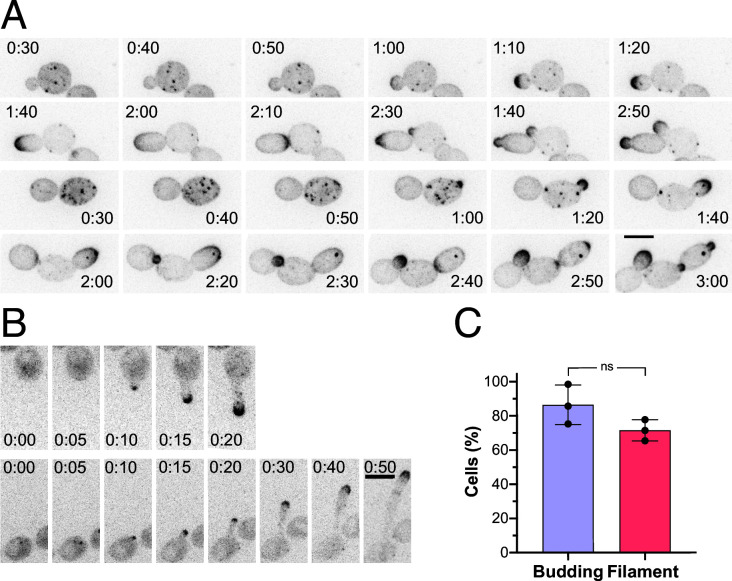
Dynamics of ergosterol during *C. albicans* budding and filamentous growth. (**A**) Ergosterol distribution is dynamic during the cell cycle. Representative time-lapse images of wild-type cells expressing RFP-D4H (PY6037) at indicated times from two time-lapse experiments are shown; images are maximum projections of 26 0.4 µm z-sections. (**B**) Ergosterol polarization persists at the filament apex. Representative images from two time-lapse experiments of wild-type cells expressing RFP-D4H (PY6037) at indicated times, grown in the presence of FBS at 37°C, are shown; images are maximum projections of 21 0.4 µm z-sections. (**C**) Ergosterol is highly polarized at the growth sites of both budding and filamentous cells. Quantitation of cells with polarized RFP-D4H signal in small buds or in filament tips (three independent experiments, with *n* = 40–80 cells per experiment). A two-tailed *t*-test revealed no significant difference (ns). Bar, 5 µm.

D4H is a 110-amino acid polypeptide that specifically binds membrane ergosterol and cholesterol. DNA encoding the D4H domain was fused to that of a codon-optimized red fluorescent protein (RFP) (17). The resulting RFP-D4H biosensor was used to probe ergosterol dynamics during budding and hyphal growth. As this biosensor is expressed in the cytoplasm, it can only bind ergosterol that is accessible to the cytoplasm, e.g., the inner leaflet of the plasma membrane. In stationary cells, as well as in cells with sub-optimal growth, punctae of RFP-D4H were observed, typically associated with the cell cortex (Fig. 1A). These punctae partially co-localized with sites of endocytosis, as visualized by the actin-binding protein 1 (Abp1) in double-labeling experiments (Fig. S1A). In *S. pombe*, sterols also accumulate in endosomal compartments (16), and in *S. cerevisiae,* esterified ergosterol is found in lipid droplets (15). Hence, we also examined whether these RFP-D4H punctae co-localized with endosomal marker Snf7 or the lipid droplet dye BODIPY and did not observe co-localization with either of these compartment markers in *C. albicans* (Fig. S1B and C). When active growth on agar pads was maintained during image acquisition, ergosterol was enriched at sites of polarized growth in *C. albicans*. Upon bud emergence, ergosterol was substantially enriched at the bud tip, concomitant with a decrease in the signal associated with punctae (Fig. 1A), and upon germ tube emergence, a tight cluster of ergosterol was observed at the apex (Fig. 1B) (17). This polarized distribution of ergosterol to sites of growth was maintained throughout the cell cycle, also appearing at cell division sites. Specifically, during either bud or germ tube emergence, ergosterol accumulated at the nascent growth site, prior to a visible protrusion. The tight cluster of ergosterol was localized to the growing tip persistently as the filament extended (17). This polarization of ergosterol at growth sites was observed in the majority (86% and 72%, respectively) of budding and filamentous cells (Fig. 1C).

In *Candida glabrata,* cholesterol uptake can occur from the host (18). Hence, we examined whether ergosterol tip enrichment was observed when *C. albicans* filamentous growth was induced in Spider media, which does not contain cholesterol. In *C. albicans,* we detected ergosterol apical enrichment in hyphae induced in Spider media; the levels of the D4H reporter at the apex were indistinguishable from that of hyphae induced with serum (Fig. S2). Together, these data indicate that highly polarized ergosterol is present in actively growing cells irrespective of cell cycle stage and growth mode.

### Polarized ergosterol distribution is not critical for growth

**Fig 2:**
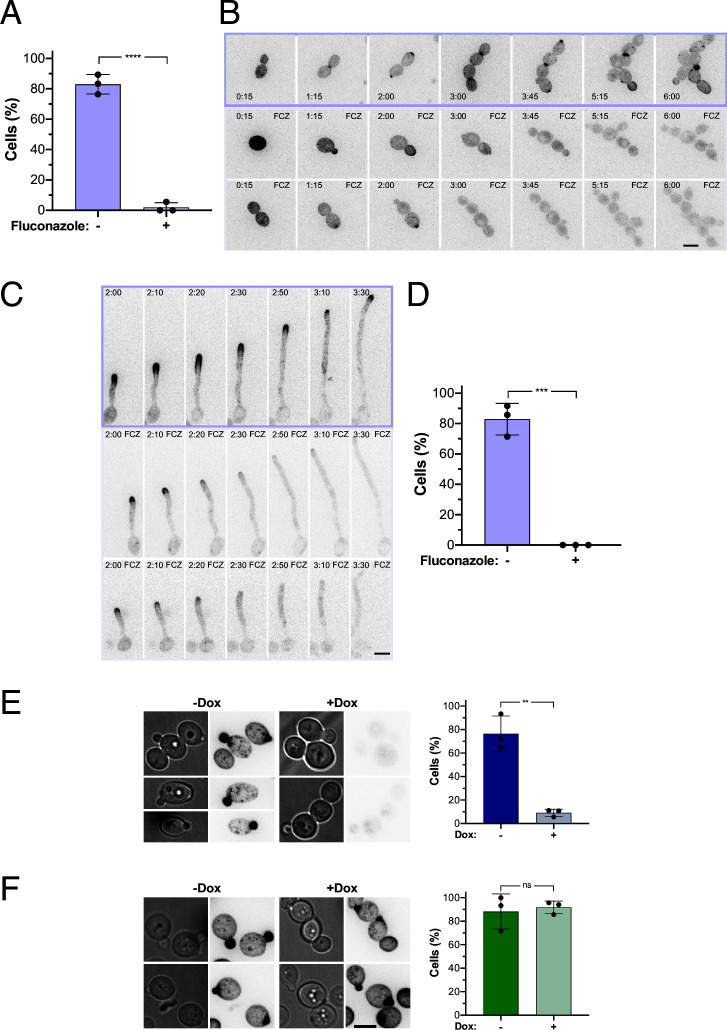
Fluconazole or repression of *ERG11* results in rapid ergosterol depletion at growth sites in *C. albicans*. (**A**) Fluconazole alters ergosterol polarization in budding cells. Wild-type cells expressing RFP-D4H (PY6037) were grown with or without 10 µg/mL fluconazole (FCZ) for 16 h; the percentage of cells with RFP-D4H signal associated with small buds is shown (three independent experiments with *n* = 10–30 cells per experiment). Error bars indicate standard deviations, and a two-tailed *t*-test revealed a significant difference, ****, *P* < 0.0001. (**B**) Ergosterol polarization is reduced upon fluconazole exposure in budding cells. Representative images from time-lapse experiments of wild-type cells expressing RFP-D4H (PY6037), grown with or without FCZ, are shown. (**C**) Ergosterol polarization is reduced upon fluconazole exposure in hyphal cells. Representative images from time-lapse experiments of wild-type cells expressing RFP-D4H (PY6037), grown in the presence of FBS with or without FCZ, are shown. (**D**) Quantitation of hyphal cells with ergosterol-enriched tip. Wild-type cells expressing RFP-D4H (PY6037) were grown with or without FCZ; the percentage of cells with RFP-D4H signal at the filament apex at 2.5-h incubation time is shown (three independent experiments, with *n* = 10–14 cells per experiment). Error bars indicate standard deviations, and a two-tailed *t*-test revealed a significant difference, ***, *P* < 0.001. (**E**) Polarized distribution of ergosterol depends on Erg11. Representative images of *erg11∆*/pTet*ERG11* cells (PY6862), grown in the absence (-Dox) or presence of doxycycline (+Dox), are shown (left panel). Quantitation of cells with a polarized RFP-D4H signal in small buds is shown (three independent experiments, *n* = 10–30 cells per experiment) (right panel). Error bars indicate standard deviations, and a two-tailed *t*-test revealed a significant difference, **, *P* < 0.01. (**F**) Polarized distribution of ergosterol does not require Erg25. Representative images of *erg25∆*/pTet*ERG25* cells (PY6859), grown as in panel E, are shown (left panel). Quantification of cells with a polarized RFP-D4H signal in small buds, as in panel E (right panel). Error bars indicate standard deviations. A two-tailed *t*-test revealed no significant difference (ns). Bar, 5 µm.

Azole drugs inhibit lanosterol demethylase, encoded by the *ERG11* gene, and lead to a reduction in ergosterol. Hence, we next examined the distribution of ergosterol upon inhibition or repression of Erg11. Either inhibition or repression of Erg11 blocks ergosterol synthesis but not sterol precursors of ergosterol. Upon incubation with a high concentration (10X MIC or above) of fluconazole (FCZ) for 16 h, essentially no cells with polarized D4H signal were observed (Fig. 2A). We verified that the RFP-D4H reporter did not substantially alter fluconazole susceptibility by growth on media containing 10 µg/mL of this drug (Fig. S3A). Interestingly, both in budding cells (Fig. 2B) and in filamenting cells (Fig. 2C and D) exposed to fluconazole for 2–3 h, ergosterol was no longer enriched at growth sites. Nonetheless, budding growth continued in the presence of these high fluconazole concentrations, with doubling times indistinguishable from that of control cells (Fig. S3B). Strikingly, within this time frame, filament extension rates were not drastically altered (Fig. S3C). Reduced filament extension rates (by ~40%) were only observed at later times (4 h). These results are consistent with inhibition of Erg11 dramatically reducing polarized ergosterol, on the order of hours.

We took a complementary approach to deplete Erg11, using a strain where the sole copy of *ERG11* was behind a doxycycline (Dox)-repressible promoter (pTet-*ERG11*). This strain had a substantial growth defect when incubated with Dox (20 µg/mL), which was not altered by the presence of RFP-D4H, and RT-PCR analyses did not detect that *ERG11* transcripts after overnight growth in Dox (Fig. S4A and B). The inhibition of pTet-*ERG11* expression by doxycycline was nearly complete, as few, if any, cells had polarized ergosterol in the presence of Dox, compared to 80% in its absence (Fig. 2E). As a control for the effect of doxycycline on D4H localization, we examined whether Dox-dependent depletion of *ERG25* altered the polarized distribution of ergosterol using a strain, in which the sole copy of *ERG25* was under the control of the repressible promoter. Adding Dox to this strain resulted in reduced *ERG25* transcript levels (Fig. S4C), yet the percentage of cells with polarized ergosterol during budding growth was similar in the absence and presence of Dox (Fig. 2F). This indicates that the D4H domain does not bind sterol precursors or aberrant sterols generated in bypass pathways, consistent with the idea that this domain specifically binds ergosterol and does not bind lanosterol (19).

In summary, our study of a specific live-cell ergosterol reporter for *C. albicans* reveals that apical enrichment of this sterol is not critical for budding and filamentous growth in this human fungal pathogen. Furthermore, it highlights that this live-cell reporter of ergosterol localization is likely to be a useful tool in the analyses of azole resistance and tolerance mechanisms, including alterations in drug targets and upregulation of efflux activities.

### Strains and media

Strains used in this study are listed in Table S1. For transformation, strains were grown in YEPD (yeast extract, peptone, dextrose) supplemented with Uridine (80 µg/mL) at 30°C. For budding growth experiments, cells were grown in synthetic complete (SC) medium, supplemented with uridine at 30°C. For filamentous growth, induction of cells was carried out on agarose pads with either 75% fetal bovine serum (FBS; PAN Biotech) in SC medium or with Spider medium at 37°C. For doxycycline (Dox) gene repression, SC was supplemented with 20 µg/mL Dox (2). For fluconazole (FCZ) experiments, cells were grown in the presence of 10 µg/mL FCZ, 0.02% dimethylsulfoxide (DMSO) or with 0.02% DMSO as a control. Lipid droplets were stained with BODIPY (Thermo Fisher Scientific ). Cells were grown overnight in SC media and incubated with 10 µM BODIPY for 30 min at room temperature prior to imaging.

The oligonucleotides used in this study are listed in Table S2. To visualize the distribution of ergosterol, we used the genetically encoded biosensor D4H with the plasmid *pExp-pACT1-mScarlet-D4H* (17). This plasmid was linearized with NcoI and integrated into the *RP10* locus. The Dox-repressible *erg11Δ/pTetERG11* and *erg25Δ/pTetERG25* strains were constructed from PY173, a derivative of BWP17 containing the tetracycline-regulatable transactivator TetR-ScHAP4AD, as described (20). The Abp1-GFP and Snf7-GFP strains were generated by homologous recombination, using pFA-GFPγ-URA (21) or pGFP-NAT1 (22) and primer pairs ABP1.P1/ABP1.P2 and SNF71.P1/SNF7.P2 , respectively.

### Microscopy and image analyses

Cells were imaged as described previously using spinning-disk confocal microscopy (17, 23) with a PLANAPO total internal reflection fluorescence (TIRF) 1.45-numerical-aperture (NA) 100× objective. All live-cell imaging time-lapses were performed starting with washed, overnight-grown cells incubated on agarose pads. To quantify D4H-associated signal intensity at growth sites, sum projections of z-stacks were analyzed using Volocity software version 6.3 (Perkin Elmer, USA), and signal was considered polarized if it was 5 or 7 standard deviations above the image mean in budding and filamentous cells, respectively. Cell doubling times were determined from time-lapse images, from bud emergence to subsequent bud emergence. D4H-associated signal intensities at filament tips were determined from exponentially grown cells incubated for 1–2 h either in Spider (24) or FBS-containing media. The apex/cytoplasm ratio of D4H signal was determined from image central z-sections, quantified with Fiji (Version 1.54 f), using regions of interest (ROIs) at the tip of the filamentous cells (apex) and 1–2 μm from this area (cytoplasm). Ratios of 1.7 or greater were scored as polarized.

### RNA extraction and RT-PCR

Cells were incubated overnight with SC media in the absence or presence of 20 µg/mL of Dox. mRNA extraction and RT-PCR were carried out as described (17). Oligonucleotide pairs ACT1.P1/ACT1.P2, ERG11.P1/ERG11.P2, and ERG25.P1/ERG25.P2 were used to amplify *ACT1*, *ERG11* and *ERG25*, respectively.

### Statistical analysis

Data were compared by unpaired *t*-test using GraphPad Prism (v. 8) software, with all *P* values indicated in figure legends.

## SUPPLEMENTAL MATERIAL

Fig. S1 mbio.02493-23-s0001.eps Cortical membrane-associated ergosterol partly co-localizes with endocytosis sites.

Fig. S2 mbio.02493-23-s0002.eps Ergosterol enrichment at the hyphal apex is independent of the hyphal growth inducer.

Fig. S3 mbio.02493-23-s0003.eps The ergosterol reporter does not alter fluconazole sensitivity, and growth is not substantially affected by short exposure to fluconazole.

Fig. S4 mbio.02493-23-s0004.eps ERG gene expression is reduced upon repression of ERG11 or ERG25.

Supplemental Legends mbio.02493-23-s0005.docx Legends for Figures S1-S4 and Movies S1-4.

Supplemental Tables mbio.02493-23-s0006.docx Tables S1 and S2.

Movie S1 mbio.02493-23-s0007.mp4 Ergosterol dynamics in budding cells.

Movie S2 mbio.02493-23-s0008.mp4 Ergosterol dynamics in filamentous cells.

Movie S3 mbio.02493-23-s0009.mp4 Effect of fluconazole on ergosterol dynamics in budding cells.

Movie S4 mbio.02493-23-s0010.mp4 Effect of fluconazole on ergosterol dynamics in filamentous cells.

ASM does not own the copyrights to Supplemental Material that may be linked to, or accessed through, an article. The authors have granted ASM a non-exclusive, world-wide license to publish the Supplemental Material files. Please contact the corresponding author directly for reuse.
