# Supplementary material for: A live-cell ergosterol reporter for visualization of the effects of fluconazole on the human fungal pathogen Candida albicans
Source: mBio. 2023 Nov 30;14(6):e02493-23. doi: 10.1128/mbio.02493-23 (PMC10746211; doi:10.1128/mbio.02493-23)
Supplement: Supplemental Legends — Legends for Figures S1-S4 and Movies S1-4. [file mbio.02493-23-s0005.docx]

**Figure S1**. **Cortical membrane associated ergosterol partly co-localizes with endocytosis sites in *C. albicans***. **A)** Ergosterol punctae partly co-localize with endocytosis sites. Overnight cultures of a strain (PY7044) expressing RFP-D4H (magenta) and Abp1-GFP (green) were imaged. Central z-sections are shown. **B)** Ergosterol punctae do not co-localize with the endosomal compartment. A strain (PY7014) expressing RFP-D4H (magenta) and Snf7-GFP (green) was imaged as in S1A and maximum projections of z-sections are shown. **C)** Ergosterol punctae do not co-localize with lipid droplets. A strain (PY6037) expressing RFP-D4H (magenta), with Bodipy labeled lipid droplets (green) was imaged as in S1A and maximum projections of z-sections are shown. Representative images from 1-3 experiments (*n* = 50-100 cells each); bar, 5 μm.

**Figure S2.** **Ergosterol enrichment at the *C. albicans* hyphal apex is independent of the hyphal growth inducer**. Representative images of wild-type cells expressing RFP-D4H (PY6037), grown in either FBS or Spider containing media at 37ºC for 90 and 120 min, respectively (left panel). Quantification of the ergosterol enrichment at the filament apex (ratio of the mean D4H apex signal to the cytoplasm; ~2 μm back the apex), *n* = 30-40 cells. Error bars indicate standard deviations. A two-tailed *t-test* revealed no significant difference (ns). Bar, 5 µm.

**Figure S3. The ergosterol reporter does not alter *C. albicans* fluconazole susceptibility, and growth is not substantially affected by short exposure to fluconazole. A)** The RFP-D4H reporter does not alter fluconazole susceptibility. Serial dilutions of indicated strains (WT, PY4861 and WT expressing RFP-D4H, PY6037) were spotted on YEPD with or without FCZ (10 µg/ml) and incubated for 3 days at 30ºC. **B)** Initial doubling time of cells is similar in the presence and absence of fluconazole. Wild-type cells expressing RFP-D4H (PY6037) were followed by time-lapse microscopy as in Fig. 2A and doubling time from *n* = 70-90 cells was determined. Error bars indicate standard deviations. A two-tailed *t-test* revealed no significant difference (ns). **C)** Initial extension rate of filamentous cells is similar in the presence and absence of fluconazole. Wild-type cells expressing RFP-D4H (PY6037) were analyzed by time-lapse microscopy as in Fig. 2C and filament length in the initial 6 times points (2:00 to 2:50) was fit with a linear regression (r^2^ > 0.95). Extension rates from *n* = 35 cells (from 3 independent experiments) was determined. Error bars indicate standard deviations. A two-tailed *t-test* revealed no significant difference (ns).

**Figure S4.** ***ERG* gene expression is reduced upon repression of *ERG11* or *ERG25* in *C. albicans*.** **A)** The RFP-D4H reporter did not alter susceptibility of the *erg11*Δ/pTet*ERG11* (PY6862) strain to doxycycline. Serial dilutions of wild-type (PY173), *erg11*Δ/pTet*ERG11* (PY6743), and *erg11*Δ/pTet*ERG11* expressing RFP-D4H (PY6862) strains were spotted on YEPD with or without Dox and incubated for 3 days at 30ºC. **B)** RT-PCR was carried out on the *erg11*Δ/pTet*ERG11* (PY6862) strain grown with or without Dox, using a CaERG11TM1 primer pair. Values are the means of determinations from three independent replicates, normalized to *ACT1* and the average level of *ERG11*/*ACT1* in the absence of Dox was set to 1. Error bars indicate standard deviations. A two-tailed *t-test* revealed a significant difference, ***, *P* < 0.001. **C)** RT-PCR was carried out on the *erg25*Δ/pTet*ERG25* (PY6859) strain as in Fig. S4B, using a CaERG25TM1 primer pair. A two-tailed *t-test* revealed a significant difference, *, *P* < 0.04.

**Movie S1.** Ergosterol dynamics in *C. albicans* budding cells. Maximum projections of RFP-D4H signal over time.

**Movie S2.** Ergosterol dynamics in *C. albicans* filamentous cells. Maximum projections of RFP-D4H signal over time in cells with FBS.

**Movie S3.** Effect of fluconazole on ergosterol dynamics in *C. albicans* budding cells. Maximum projections of RFP-D4H signal over time in cells grown with or without 10 µg/ml FCZ.

**Movie S4.** Effect of fluconazole on ergosterol dynamics in *C. albicans* filamentous cells. Maximum projections of RFP-D4H signal over time in cells grown in FBS with or without 10 µg/ml FCZ.
