# Supplementary material for: A live-cell ergosterol reporter for visualization of the effects of fluconazole on the human fungal pathogen Candida albicans
Source: mBio. 2023 Nov 30;14(6):e02493-23. doi: 10.1128/mbio.02493-23 (PMC10746211; doi:10.1128/mbio.02493-23)
Supplement: Supplemental Tables — Tables S1 and S2. [file mbio.02493-23-s0006.docx]

Table S1. Strains used in this study.

| **Strain number** | **Relevant Genotype** | **Source** |
| --- | --- | --- |
| BWP17 | *ura3Δ::λimm434/ura3Δ::λimm434 his1Δ::hisG/his1Δ::his arg4::hisG/arg4Δ::hisG* | (25) |
| PY173 | Same as BWP17 with *ENO1/eno1::ENO1-tetR ScHAP4AD- 3xHA-ADE2* | (20) |
| PY6037 | Same as BWP17 with *RP10::ARG4-pAct1-mSc-D4H* | (17) |
| PY6659 | Same as PY173 with *erg25Δ::HIS1* | This study |
| PY6661 | Same as PY173 with *erg11::URA3pTet_off_ERG11* | This study |
| PY6689 | Same as PY6659 with *erg25::URA3pTet_off_ERG25* | This study |
| PY6743 | Same as PY6661 with *erg11Δ::HIS1* | This study |
| PY6859 | Same as PY6689 with *RP10::ARG4-pAct1-mSc-D4H* | This study |
| PY6862 | Same as PY6743 with *RP10::ARG4-pAct1-mSc-D4H* | This study |
| PY7014 | Same as PY6037 with *ABP1::SNF7-GFP-SAT1* | This study |
| PY7044 | Same as PY6037 with *ABP1::ABP1-GFP-URA3* | This study |

Table S2. Oligonucleotides used in this study.

| **Oligonucleotide number** | **Sequence (5’ to 3’)** |
| --- | --- |
| CaErg11pTetOKin | TAAATAGACAAAGAAAGGGAATTCAATCGTTATTCTTTCCATATTACTTGTCTTCTTTTTATTATATATATAAGTTTCTTTTaggaattgatttggatgg |
| CaErg11mTetOKin | TAATATACTGATCTGTTGTGTAACACTAAGGGACAAAAAATAATTAATGCCATCAATGACAGTTTCAACAATAGCCATctagttttctgagataaagct |
| CaErg11.S1 | AAACAAAAAAATAAAAAATAATATCATACAATAATTTTTCTTCATCTTACTTCTTTCTTTCAATCTTTAATAAATCAATTgaagcttcgtacgctgcaggtc |
| CaErg11.S2 | TATGTGTATATATGTTAATCCAACTAAGTAACAAAATGAAAACAATCTGAACACTGAATCGAAAGAAAGTTGCCGTTTTAtctgatatcatcgatgaattcgag |
| CaErg25pTetOKin | GATTCATTAATTGTTATATTTCAACATATACATATTCATTTATTCCTTGATCCTTTTTTAAAGTATTCAATTTATTTATTTAaggaattgatttggatgg |
| CaErg25pTetOKin | ATTTTGATAAACTTGGGAAAAAGTAGTTGCATTCAGAAAACTCGAATAGTCATGATAAACATTACTAATGGAAGACATctagttttctgagataaagctg |
| CaErg25.S1 | TTTTTTCTATTCTTTCTTTGCTTTTGTTAAGGGCATTGCTTTTCTTTTATATTAACTCATAATTTCATTCTTTTGATTCAgaagcttcgtacgctgcaggtc |
| CaErg25.S2 | ATTAAATAAAACCAAAAATAAACAGCATCAATATTGAAAGTGGTACAAACATTGAGAAGTTGTACACATGTACTCTCTCTtctgatatcatcgatgaattcgag |
| CaAbp1XFP.S1 | TGTTGAAATCGAATTTGTTGACGATGATTGGTGGCAAGGAAAACATTCCAAGACAGGAGAAGTCGGATTGTTCCCTGCTAACTATGTTGTCTTGAATGAGggtgctggcgcaggtgcttc |
| CaAbp1.S2 | CAATTTATCTTTTCTTTGTATTTATATTATAGATTCATATAAAAAAAAAACGAATATTGTTTATACTAAATtctgatatcatcgatgaattcgag |
| CaSnf7XFP.S1 | CTGTTAACAAGAAAGCTCCAGTAGTAGAAGAAGATGAAGATGAAGAAGCATTGAAAGCATTGCAAGCTGAAATGGGATTAggtggtggttctaaaggtgaag |
| CaSnf7XFP.S2 | CAAAAAACAGGGGCAAAGAAAAGGTAATATATGTTTCTATACAAAGCTTTCGTTATTCTCCGTATTCGGTATTTCAAACAcgttagtatcgaatcgacagc |
| ACT1pTM | atgttcccaggtattgctga |
| ACT1pTM | acatttgtggtgaacaatgg |
| ERG11pTM | TGACCGTTCATTTGCTCAACTATATT |
| ERG11mTM | CACGTTCTCTTCTCAGTTTAATTTCTTTC |
| ERG25pTM | AAGATTATTCCAAGCCGTTGATG |
| ERG25mTM | CCACCAATGAAATAATGATGATGTTC |

**Reference**

1. Wilson RB, Davis D, Mitchell AP. 1999. Rapid hypothesis testing with Candida albicans through gene disruption with short homology regions. J Bacteriol 181:1868–1874. https://doi.org/10.1128/JB.181.6.1868-1874.1999.
